# Tamiflu Swan Song?: Building Resistance to Top Avian Flu Drug

**Published:** 2007-01

**Authors:** Cynthia Washam

As the WHO has begun warning of the potential for an avian flu pandemic, governments worldwide have been stockpiling Tamiflu (oseltamivir phosphate). Tamiflu minimizes flu symptoms and duration by preventing the virus from escaping the cells it infects. It also reduces the likelihood of spreading the virus. Now British researchers are predicting that heavy use of Tamiflu, as during a pandemic, will expose wild waterfowl to enough of the antiviral agent to foster a resistant strain **[*EHP* 115:102–106; Singer et al.]**.

The risk that Tamiflu will promote a resistant virus comes from the drug’s excreted metabolite, oseltamivir carboxylate (OC), which is in fact the active antiviral. Up to 80% of ingested Tamiflu is excreted as OC in urine and feces. OC withstands degradation through sewage treatment and for several weeks afterward. Birds drinking water from catchments contaminated with OC would ingest the antiviral, which would inhibit nonresistant viruses in the birds’ digestive systems while enabling resistant viruses to proliferate. Birds excreting the resistant virus would spread the strain among other waterfowl at the same body of water.

To estimate birds’ exposure to OC, Singer and his colleagues examined data on OC’s biodegradability along with measurements of wastewater discharges into 16 major catchment areas in the United States and the United Kingdom. They estimated the number of flu cases in an outbreak within each catchment. Among other suppositions, the researchers assumed that all cases were treated with a standard five-day regimen of Tamiflu.

The team calculated that the most vulnerable catchment in the United States is the Lower Colorado, where they predicted OC concentrations high enough to promote Tamiflu resistance in the virus for up to eight weeks. The most vulnerable British catchment would be the Lee catchment in northeast London. Resistant strains could proliferate within a week after pandemic starts in a region, assuming all patients start taking Tamiflu as soon as they develop symptoms. The authors also note that the range of predicted concentrations could have yet-uncharacterized ecotoxicologic effects.

Singer and colleagues call for more detailed modeling of OC water contamination, particularly in Asia, where the virus is most prevalent and human-to-wildfowl contact is more common. They also recommend studies of ways to minimize the release of OC into waterways, which could include biological and chemical pretreatment in the toilet bowl.

## Figures and Tables

**Figure:**
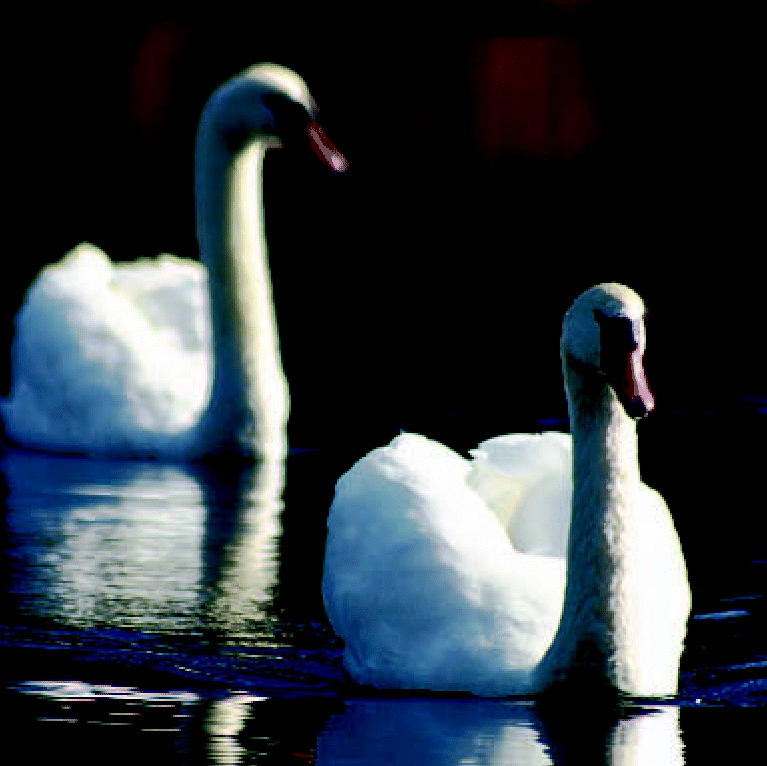
Release and catch. Release of excreted Tamiflu into the environment could create drug-resistant strains of avian flu in wild waterfowl.

